# Inhibition and Induction by Poziotinib of Different Rat Cytochrome P450 Enzymes *In Vivo* and in an *In Vitro* Cocktail Method

**DOI:** 10.3389/fphar.2020.593518

**Published:** 2021-01-05

**Authors:** Jinhui Wang, Feifei Chen, Hui Jiang, Jia Xu, Deru Meng, Peiwu Geng, Dapeng Dai, Jingbo Hu, Yunfang Zhou, Quan Zhou, Shuanghu Wang

**Affiliations:** ^1^ Institute of Drug Discovery Technology, Ningbo University, Ningbo, China; ^2^ The Laboratory of Clinical Pharmacy, The Sixth Affiliated Hospital of Wenzhou Medical University, The People’s Hospital of Lishui, Lishui, China; ^3^ The Key Laboratory of Geriatrics, Beijing Institute of Geriatrics, Beijing Hospital, National Center of Gerontology, National Health Commission, Institute of Geriatric Medicine, Chinese Academy of Medical Sciences, Beijing, China

**Keywords:** pozioitinib, cancer, drug-drug interactions, inhibitor, cytochrome P450, cocktail method, pharmacokinetics

## Abstract

Poziotinib is an orally active, irreversible, pan-HER tyrosine kinase inhibitor used to treat non-small cell lung cancer, breast cancer, and gastric cancer. Poziotinib is currently under clinical investigation, and understanding its drug-drug interactions is extremely important for its future development and clinical application. The cocktail method is most suitable for evaluating the activity of cytochrome P450 enzymes (CYPs). As poziotinib is partially metabolized by CYPs, cocktail probes are used to study the interaction between drugs metabolized by each CYP subtype. Midazolam, bupropion, dextromethorphan, tolbutamide, chlorzoxazone, phenacetin, and their metabolites were used to examine the effects of poziotinib on the activity of cyp1a2, 2b1, 2d1, 2c11, 2e1, and 3a1/2, respectively. The *in vitro* experiment was carried out by using rat liver microsomes (RLMs), whereas the *in vivo* experiment involved the comparison of the pharmacokinetic parameters of the probes after co-administration with poziotinib to rats to those of control rats treated with only probes. UPLC-MS/MS was used to detect the probes and their metabolites in rat plasma and rat liver microsomes. The *in vitro* results revealed that the half-maximal inhibitory concentration values of bupropion and tolbutamide in RLMs were 8.79 and 20.17 μM, respectively, indicating that poziotinib showed varying degrees of inhibition toward cyp2b1 and cyp2c11. Poziotinib was a competitive inhibitor of cyp2b1 and cyp2c11, with Ki values of 16.18 and 17.66 μM, respectively. No time- or concentration-dependence of inhibition by poziotinib was observed toward cyp2b1 and cyp2c11 in RLMs. Additionally, no obvious inhibitory effects were observed on the activity of cyp1a2, cyp2d1, cyp2e1, and cyp3a1/2. *In vivo* analysis revealed that bupropion, tolbutamide, phenacetin, and chlorzoxazone showed significantly different pharmacokinetic parameters after administration (*p* < 0.05); there was no significant difference in the pharmacokinetic parameters of dextromethorphan and midazolam. These results show that poziotinib inhibited cyp2b1 and cyp2c11, but induced cyp1a2 and cyp2e1 in rats. Thus, poziotinib inhibited cyp2b1 and cyp2c11 activity in rats, suggesting the possibility of interactions between poziotinib and these CYP substrates and the need for caution when combining them in clinical settings.

## Introduction

Drug-drug interaction (DDI) occurs during the co-administration of two or more drugs and may increase toxicity or weaken the therapeutic effect of the drugs, leading to the occurrence of serious clinical complications such as adverse drug reactions or treatment failure (Lee et al., 2015; Chen Z. H. et al., 2016). DDI is believed to be closely linked to the inhibition or induction of the activity of cytochrome P450 (CYP) enzymes because CYP enzymes play major roles in the metabolism of various drugs (Polasek et al., 2011; Wang et al., 2019). As multi-drug combination therapy is a common therapeutic strategy, the risk of CYP inhibition or induction is greater for drugs with a narrow therapeutic window, with even a small change in concentration causing toxicity or therapeutic failure of the drug (Guidoni et al., 2016). Therefore, it is necessary to understand potential DDIs in the early stages of drug development.

CYP is a superfamily of oxygenases with mixed functions that are involved in the metabolism of endogenous and exogenous substances and are abundantly present in the liver and intestines (Wang et al., 2014; Yin et al., 2016). CYPs are responsible for ∼75% of human drug metabolism and play an important role in the elimination of commonly used drugs (Carrão et al., 2018; Jo et al., 2018). Among the many CYPs, CYP3A, CYP1A, CYP2B, CYP2C, and CYP2D play important roles in drug metabolism. CYP3A4 is the most important isoenzyme subfamily comprising 40% of all CYPs and metabolizing ∼50% of drugs such as immunosuppressants, antibiotics, statins, and herbal drugs (Loue and Tod, 2014; Wang S. H. et al., 2015; Liu et al., 2017; Wang et al., 2017; Lu et al., 2018).

CYP2C is the second most abundant isoenzyme subfamily. Its main isoenzymes are CYP2C9 and CYP2C19 can metabolize ∼25% of all drugs, of which CYP2C9 metabolizes 16% of all drugs, e.g., oral anticoagulants, non-steroidal anti-inflammatory drugs, various fatty acids, and steroid hormones (Cheng et al., 2013; Dai et al., 2014; Sun et al., 2014; Zhang et al., 2018). CYP1A2 comprises ∼13% of all CYPs and metabolizes several commonly used drugs such as theophylline, tacrine, and even some important endogenous compounds (Ragia et al., 2014; Wang X. S. et al., 2015). CYP2D6 (∼2% of all CYPs) metabolizes ∼20% of all marketed drugs (Tracy et al., 2016). CYP2E1 (∼10% of all CYPs) plays an important role in the metabolism of alcohol, organic solvents, and carcinogens (Ye et al., 2015; Xu et al., 2017; Jin et al., 2018). Unlike other CYPs, CYP2B6 does not play a major role in drug metabolism, but is known to metabolize bupropion and methadone and is believed to increase the risk of breast cancer (Justenhoven et al., 2014; Yuce-Artun et al., 2014; Grangeon et al., 2017).

The cocktail method, involving the simultaneous use of several probe substrates, has been used to investigate DDIs and examine changes in CYP activity in drug metabolism (Spaggiari et al., 2016). The FDA recommends the study of DDIs for seven main isoenzymes—CYP1A2, CYP2B6, CYP2C8, CYP2C9, CYP2C19, CYP2D6 and CYP3A4. Although the CYP2E1 substrate, chlorzoxazone, is not commonly used as a cocktail probe because both chlorzoxazone and its metabolites are mainly detected in the negative ion mode of electrospray ionization (ESI), many studies have included it as a component of their cocktail approach (De Andrés et al., 2014; Tanaka et al., 2014).

Poziotinib (HM781-36B) is a new, orally active, irreversible pan-HER tyrosine kinase inhibitor (Kim et al., 2013). Poziotinib is currently undergoing clinical trials for various indications such as non-small cell lung cancer, breast cancer, and gastric cancer. Common adverse reactions caused by poziotinib treatment include rash, diarrhea, and itching (He et al., 2019; Kim T. Y. et al., 2019). In addition, in recent years, studies have found that poziotinib has a therapeutic effect on lung cancer with *EGFR* and *HER2* exon 20 mutations (Koga et al., 2018). Analyses of its metabolites by LC-MS/MS reveal that its two main metabolites, M1 (dihydroxylation) and M2 (demethylation), are mainly produced by CYP3A4 and CYP2D6 activity (Kim et al., 2013). This evidence shows that poziotinib is mainly metabolized by the liver; however, the effects of poziotinib on CYP450 isoenzymes have not been reported.

It is extremely important to select the appropriate probe substrate for each CYP450 enzyme. Studies reporting the cocktail probe approach have used phenacetin for CYP1A2, bupropion for CYP2B6, tolbutamide for CYP2C9, dextromethorphan for CYP2D6, chlorzoxazone for CYP2E1, and midazolam for CYP3A4 (Palmer et al., 2001; Spaggiari et al., 2014; Giri et al., 2019; Kim H. J. et al., 2019; Chen A. et al., 2016). Therefore, we aimed to investigate the interaction of poziotinib with a cocktail of midazolam, bupropion, dextromethorphan, tolbutamide, chlorzoxazone, and phenacetin and examine their metabolites in rat plasma by UPLC-MS/MS, and compare the effects of poziotinib on cyp1a2, cyp2b1, cyp2c11, cyp2d1, cyp2e1, and cyp3a1/2 *in vivo and in vitro*. The results of this study will help to understand the potential drug interactions of poziotinib.

## Materials and Methods

### Chemicals and Reagents

Poziotinib (purity > 98%) was purchased from the Beijing Sunflower and Technology Development Co. Ltd. (Beijing, China). Bupropion hydrochloride (purity > 98%) and phenacetin (purity > 99%) were purchased from TCI Chemicals (Shanghai, China). Tolbutamide (purity > 97.5%) and chlorzoxazone (purity > 98%) were purchased from J&K Chemical Ltd. (Shanghai China). α-Naphthoflavone (purity > 98%), Ticlopidine (purity > 98%), sulfaphenazole (purity > 98%), quinidine (purity > 98%), sodium diethyldithiocarbamatre (purity > 97%), ketoconazole (purity > 99%) were purchased from Aladdin. Midazolam injection was obtained from Nhwa Pharma. Co. (Jiangsu, China) and NADPH from Roche (Mannheim, Germany). The internal standard (IS) of diazepam (purity > 98%) was purchased from the Tianjin KingYork Pharmaceutical Co. Ltd. (Tianjin, China). Chromatographic grade methanol and acetonitrile were purchased from Merck Co. Ltd. (Darmstadt, Germany). Rat liver microsomes (RLMs) were prepared in our laboratory (Zhou et al., 2020). All other chemicals were of analytical grade or higher.

### Instruments and Conditions

Samples were analyzed by a UPLC-MS/MS system equipped with a triple quadrupole mass spectrometer (Waters Corp., Milford, MA, United States) and Acquity UPLC BEH C18 column (1.7 μm, 2.1 × 50 mm) at 40°C. The mobile phase consisted of acetonitrile (A) and 0.1% formic acid (B) with a linear gradient elution at 0.4 ml/min for 3 min. The elution procedure was as follows: 0.0–0.6 min (rapidly rising from 10 to 50% A), 0.6–1 min (increasing from 50 to 80% A), 1–2 min (increasing from 80 to 95% A), 2–2.5 min (maintained at 95% A), and 2.5–2.6 min (reduced to 10% A).

Mass spectrometer contained a triple quadrupole and an ESI source. The optimized parameters of multiple reaction monitoring detection included a source temperature of 150°C and desolvation temperature of 500°C. The MS conditions, chemical structure, and chromatograms for the probe drugs and IS are presented in Table 1 and in Supplementary Figures S1, S2 (Wang et al., 2020). All sample data were acquired with MassLynx 4.1 software (Waters Corp.).

**TABLE 1:** Mass spectrometric parameters for six substrate probes, their metabolites, and diazepam.

Compounds	Mass transition (m/z)	Ion mode	Cone voltage (V)	Collision energy (eV)
Phenacetin	180.1→109.9	Positive	35	15
4-Acetamidophenol	152.0→110.0	Positive	35	20
Bupropion	240.1→184.1	Positive	24	12
Hydroxy bupropion	256.0→238.2	Positive	20	12
Dextromethorphan	272.2→147.0	Positive	45	30
*O*-demethyl dextromethorphan	258.1→157.1	Positive	50	36
Tolbutamide	271.2→155.1	Positive	30	15
Hydroxy tolbutamide	287.0→107.0	Positive	30	26
Midazolam	326.0→291.0	Positive	50	28
Hydroxy midazolam	342.0→324.0	Positive	40	22
Chlorzoxazone	168.1→132.0	Negative	48	20
6-Hydroxy chlorzoxazone	184.1→120.0	Negative	36	20
Diazepam	285.1→193.1	Positive	35	30

### Half-Maximal Inhibitory Concentration Determination Using the Cocktail Method

The cocktail method was employed to analyze the effects of poziotinib on cyp1a2, cyp2b1, cyp2c11, cyp2d1, cyp2e1, and cyp3a1/2. First, 100 μM poziotinib was used to test its direct inhibitory effects RLMs. Then half-maximal inhibitory concentration (IC_50_) of poziotinib was determined with all of the CYPs. Phenacetin, bupropion, tolbutamide, dextromethorphan, chlorzoxazone, and midazolam (40, 20, 100, 10, 40, and 5 μM, respectively) were mixed to form a cocktail of probe substrates for measuring CYP450 activity. To this cocktail, 1 mg/ml of RLMs, 100 mM potassium phosphate buffer (pH 7.4), poziotinib (1, 2.5, 5, 10, 25, 50, or 100 μM) were added to obtain a final volume of 200 μL. After pre-incubation of this mixture for 5 min in a shaking water bath at 37°C, 1 mM NADPH was added to start the reaction. The reactions were allowed to proceed for 30 min, after which 200 μL of acetonitrile and 20 μL of IS were added to terminate the reactions. The mixtures were vortexed for 30 s and centrifuged again at 13,000 rpm for 5 min. Then, 2 μL of the supernatant was injected into the UPLC-MS/MS system for analysis.

### Mechanism of Inhibition of cyp2b1 and cyp2c11 by Poziotinib

To elucidate the mechanism of inhibition of cyp2b1 and 2c11 by poziotinib, we examined the interaction of different concentrations of poziotinib with bupropion and tolbutamide, respectively. Poziotinib was preincubated with 1 mg/ml of RLMs and probe substrate in a 37°C shaking water bath for 5 min, following which NADPH was added and incubated for 30 min. The concentrations of poziotinib were 0, 2, 4, 8 and 16 μM for 2b1, 0, 5, 10 20 and 40 μM for 2c11. The concentrations of bupropion were 5, 10, 20, and 40 μM, and the concentrations of tolbutamide were 25, 50, 100, and 200 μM. Poziotinib was incubated with different concentrations of the probe substrates in triplicate. The reactions were carried out, stopped, and processed as described in 2.3. Lineweaver-Burk plots were graphed.

### Time- and Concentration-dependent Inhibition of cyp2b1 and cyp2c11

A mixture containing 1 mg/ml of RLMs, different concentrations of poziotinib (0, 2, 4, 8 and16 μM for 2b1; 0, 5, 10 20 and 40 μM for 2c11), and NADPH was pre-incubated at 37°C for 0, 5, 10, and 20 min; Methanol instead of poziotinib was performed as control. Then 20 μM bupropion or 100 μM tolbutamide was added and incubated at 37°C for 30 min. The reactions were carried out and analyzed in triplicate as described in 2.3.

### Animal Experiments

Male Sprague-Dawley rats were supplied by the Animal Experimental Center of the Wenzhou Medical University (Wenzhou, China). Animals were kept under standard conditions with a 12-h day-night cycle and were provided water and food *ad libitum* for 2 weeks for acclimation. All of the experimental procedures followed the guidelines for the care and use of laboratory animals and were approved (No. wydw 2019-650) by the Animal Experimental Ethical Inspection of Laboratory Animal Center, Wenzhou Medical University.

### Effects of Poziotinib on the Pharmacokinetic of Substrates *In Vivo*

Twelve male Sprague-Dawley rats (230 ± 20 g) were randomly divided into treated and control groups, with 6 in each group. Dose of probe substrate mixture (phenacetin, bupropion, midazolam, chlorzoxazone and dextromethorphan) was dissolved in 0.5% CMC-Na solution: 10 mg/kg, Toluamide: 1 mg/kg. After 7 days of continuous oral administration of poziotinib (5 mg/kg, dissolved in 0.5% CMC-Na solution) to the experimental group, the two group rats were treated with an oral gavage of probe substrate mixture. After 0.083, 0.25, 0.5, 1, 2, 3, 4, 6, 8, 12, and 24 h following administration of poziotinib, 300 μL blood was collected from the tail vein of each rat, transferred to a heparinized centrifuge tube, and immediately centrifuged at 4,000 rpm for 10 min. The supernatant was mixed with 200 μL of acetonitrile and 20 μL of 0.5 μg/ml diazepam (IS), vortexed for 30 s, centrifuged at 13,000 rpm for 5 min, and the supernatant was injected into the UPLC-MS/MS system for analysis.

### Statistical Analysis

Pharmacokinetic parameters were calculated using DAS software (Version 3.2.8). SPSS software (version 16.0; SPSS Inc., Chicago, IL, United States) was used to analyze the significance of differences between the main pharmacokinetic parameters. *p* < 0.05 was considered statistically significant. GraphPad (version 8.0; GraphPad Software Inc., San Diego, CA, United States) was used to calculate IC_50_ and plot the plasma concentration-time curves and inhibition plots (type of inhibition, time- and concentration-dependence).

## Results

### Half-Maximal Inhibitory Concentration Determination Using Cocktail Method

**FIGURE 1:**
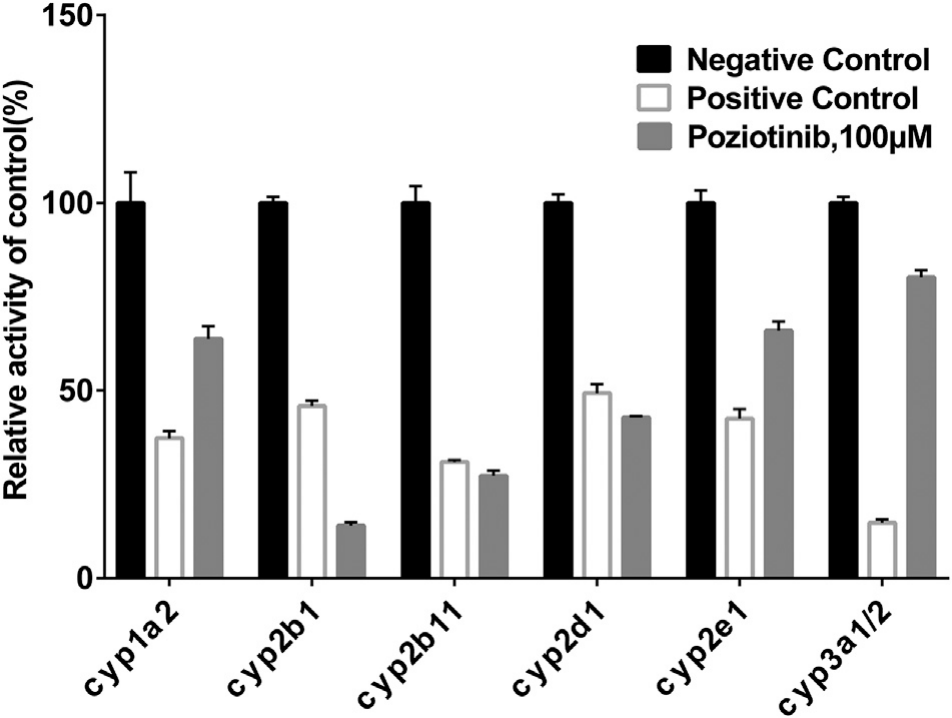
Inhibition of poziotinib on rat liver microsome. The concentrations of positive control inhibitors were as follows: 50 μM α-Naphthoflavone for cyp1a2, 50 μM Ticlopidine for cyp2b1, 20 μM sulfaphenazole for cyp2c11, 10 μM quinidine for cyp2d1, 100 μM sodium diethyldithiocarbamatre for cyp2e1, 1 μM ketoconazole for cyp3a1/2. Poziotinib was 100 μM. All data were mean ± SD of triplicate determinations.

**FIGURE 2:**
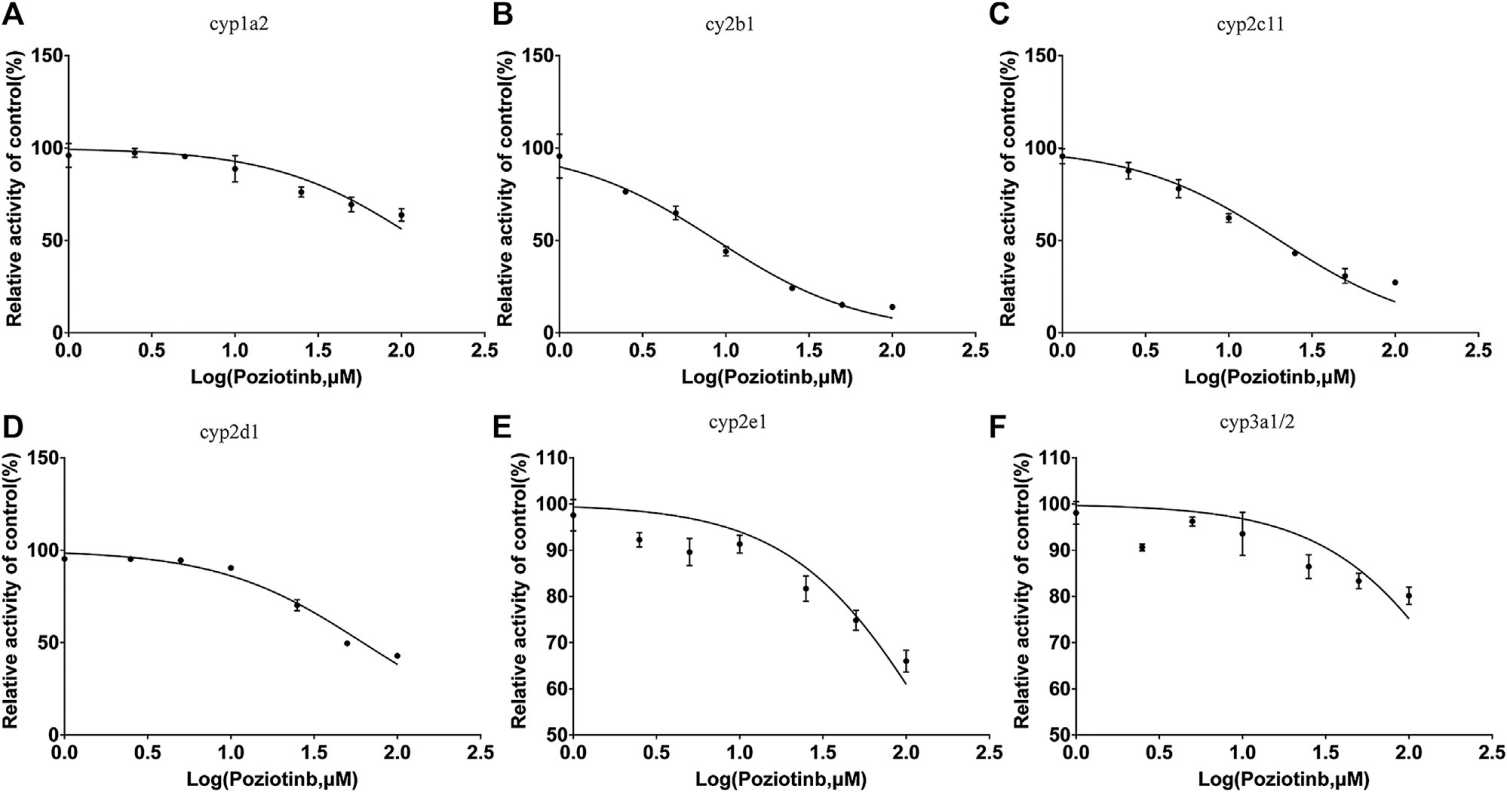
Determination of half-maximal inhibitory concentrations (IC_50_) of poziotinib using **(A)** 40 μM phenacetin for cyp1a2, **(B)** 20 μM bupropion for cyp2b1, **(C)**100 μM tolbutamide for cyp2c11, **(D)** 10 μM dextromethorphan for cyp2d1, **(E)** 40 μM chlorzoxazone for cyp2e1, and **(F)** 5 μM midazolam for cyp3a1/2 in rat liver microsomes. All data were mean ± SD of triplicate determinations.

To examine the effects of poziotinib on the six CYPs, different concentrations of poziotinib and the cocktail of all probe substrates were incubated with RLMs to determine the IC_50_ values of the six probe substrates (Figure 1). The IC_50_ values of bupropion, tolbutamide, dextromethorphan, phenacetin, chlorzoxazone and midazolam in RLMs were 8.79, 20.17, 61.96, 128.2, 156.3, and 302.7 μM respectively. Poziotinib showed weak inhibition toward cyp2d1 (Figure 2D). In contrast, the inhibitory effects of poziotinib on cyp2b1 and 2c11 were strong (Figures 2B,C). Poziotinib did not show any obvious inhibitory effect on cyp1a2 (Figure 2A), cyp2e1 (Figure 2E) or CYP3a1/2 (Figure 2F). As these results showed that pozitinib could significantly inhibit the activities of cyp2b1 and 2c11 *in vitro*.

### Mechanism of Inhibition of cyp2b1 and cyp2c11 by Poziotinib

**FIGURE 3:**
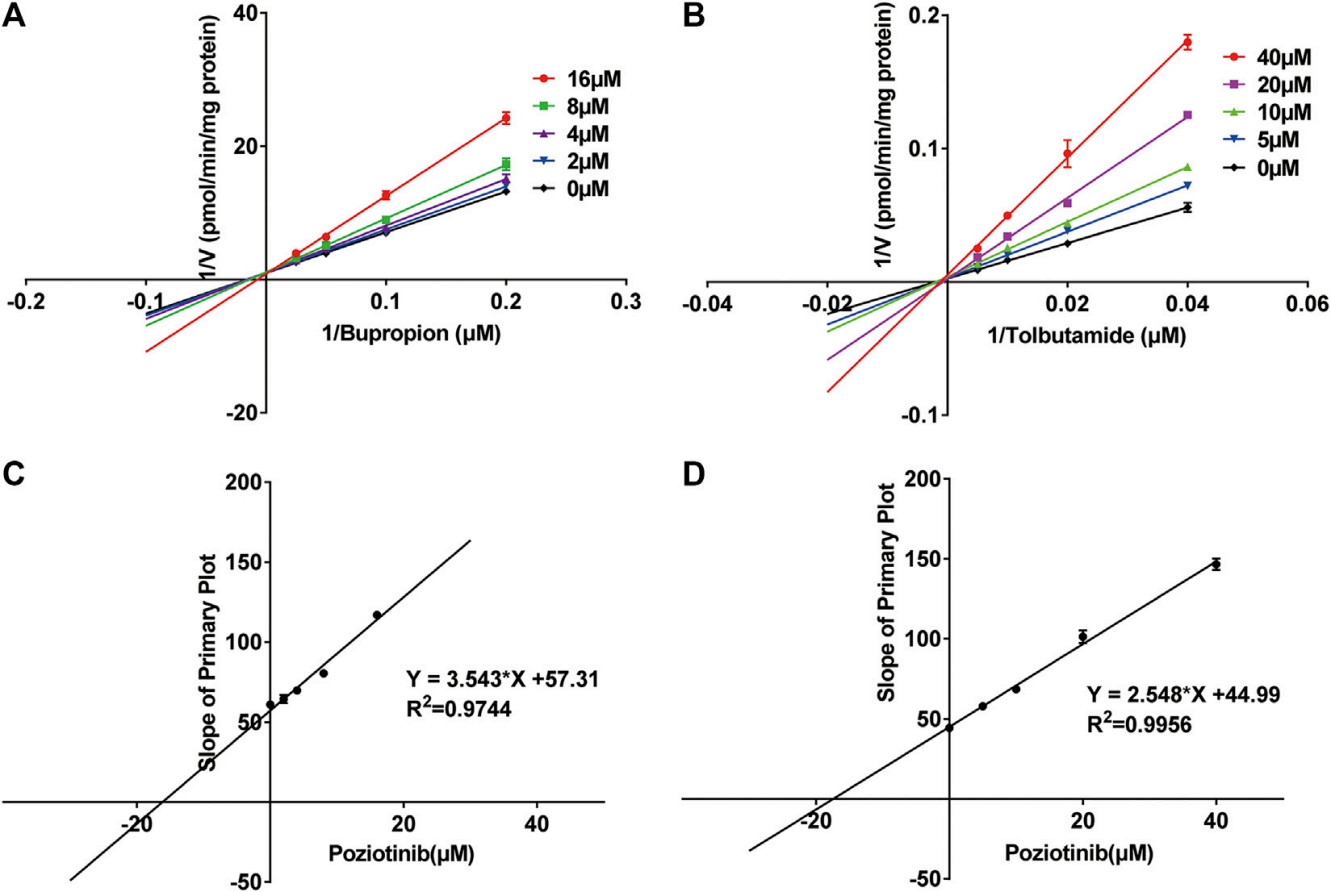
Primary Lineweaver–Burk plots of poziotinib (0, 2, 4, 8, 16 μM for bupropion, and 0, 5, 10, 20, 40 μM for tolbutamide) inhibited **(A)** bupropion (5, 10, 20 and 40 μM) and **(B)** tolbutamide (25, 50, 100 and 200 μM); Secondary plots for determination of Ki of **(C)** bupropion and **(D)** tolbutamide. All data were mean ± SD of triplicate determinations.

**FIGURE 4:**
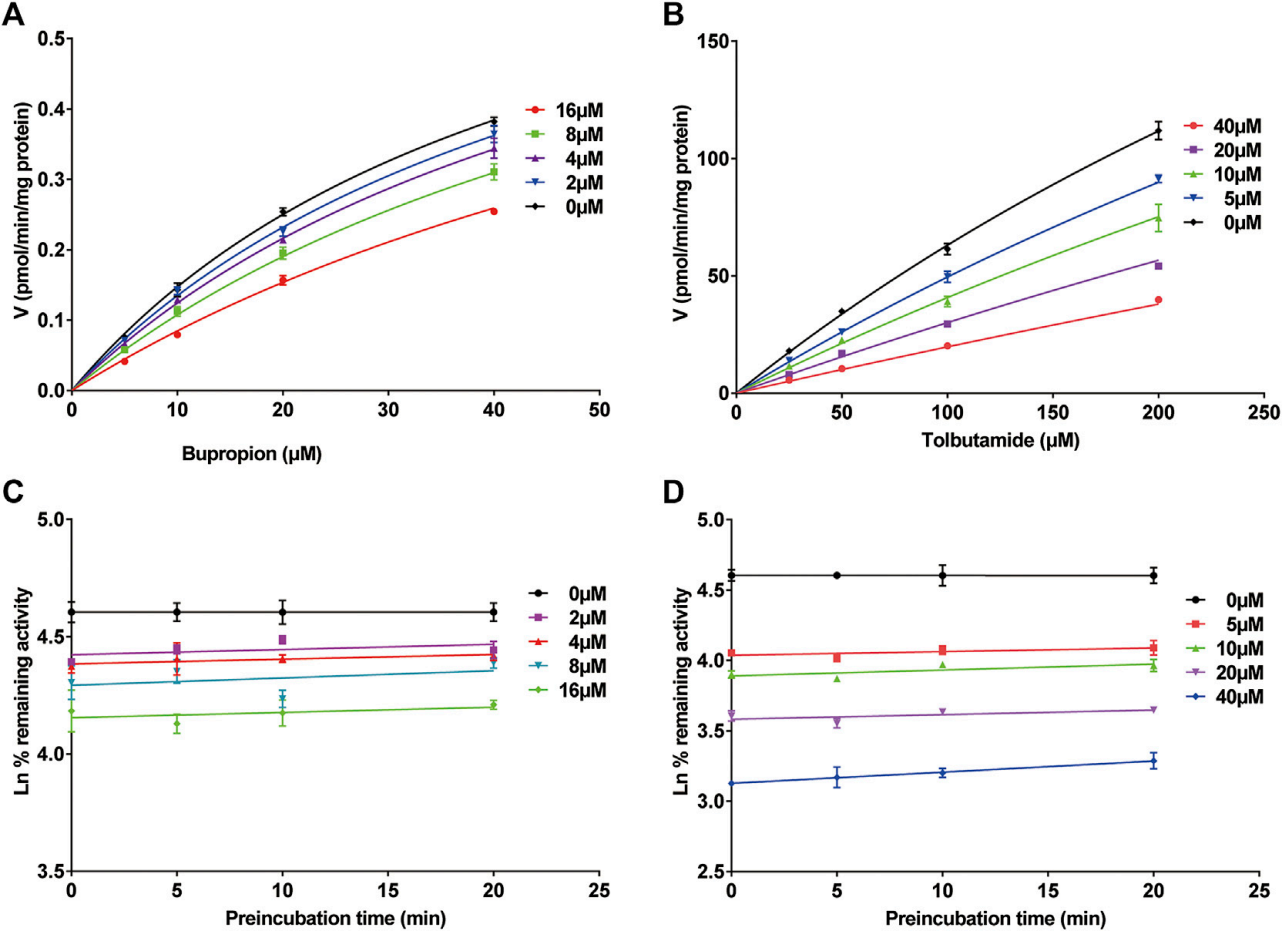
Michaelis-Menten saturation curves of **(A)** bupropion and **(B)** tolbutamide. Time- and concentration-dependent inhibition of poziotinib on rat liver microsome. Incubation reaction mixtures were pre-incubated for 0, 5, 10 and 20 min without or with poziotinib at 2, 4, 8 and 16 µM for bupropion **(C)** or 5, 10, 20, 40 µM for tolbutamide **(D)**. All data were mean ± SD of triplicate determinations.

We investigated the mechanism of the inhibitory effects of poziotinib on cyp2b1 and cyp2c11 activity by constructing Lineweaver-Burk plots (Figures 3A,B) and Michaelis-Menten saturation curves (Figures 4A,B). The Lineweaver–Burk plots show that all the straight lines intersect at a point on the *Y* axis (Figures 3A,B), indicating that the inhibition of 2b1 and 2c11 by poziotinib was competitive, with Ki values of 16.18 and 17.66 μM, respectively (Figures 3C,D).

### Time- and Concentration-dependent Inhibition of cyp2b1 and cyp2c11

The inhibition of cyp2b1 and 2c11 by poziotinib was reversible, and poziotinib had no time- or concentration-dependent inhibitory effects on cyp2b1 and cyp2c11 (Figures 4C,D).

### Effects of Poziotinib on the Activity of cyp1a2 in Rats

**FIGURE 5:**
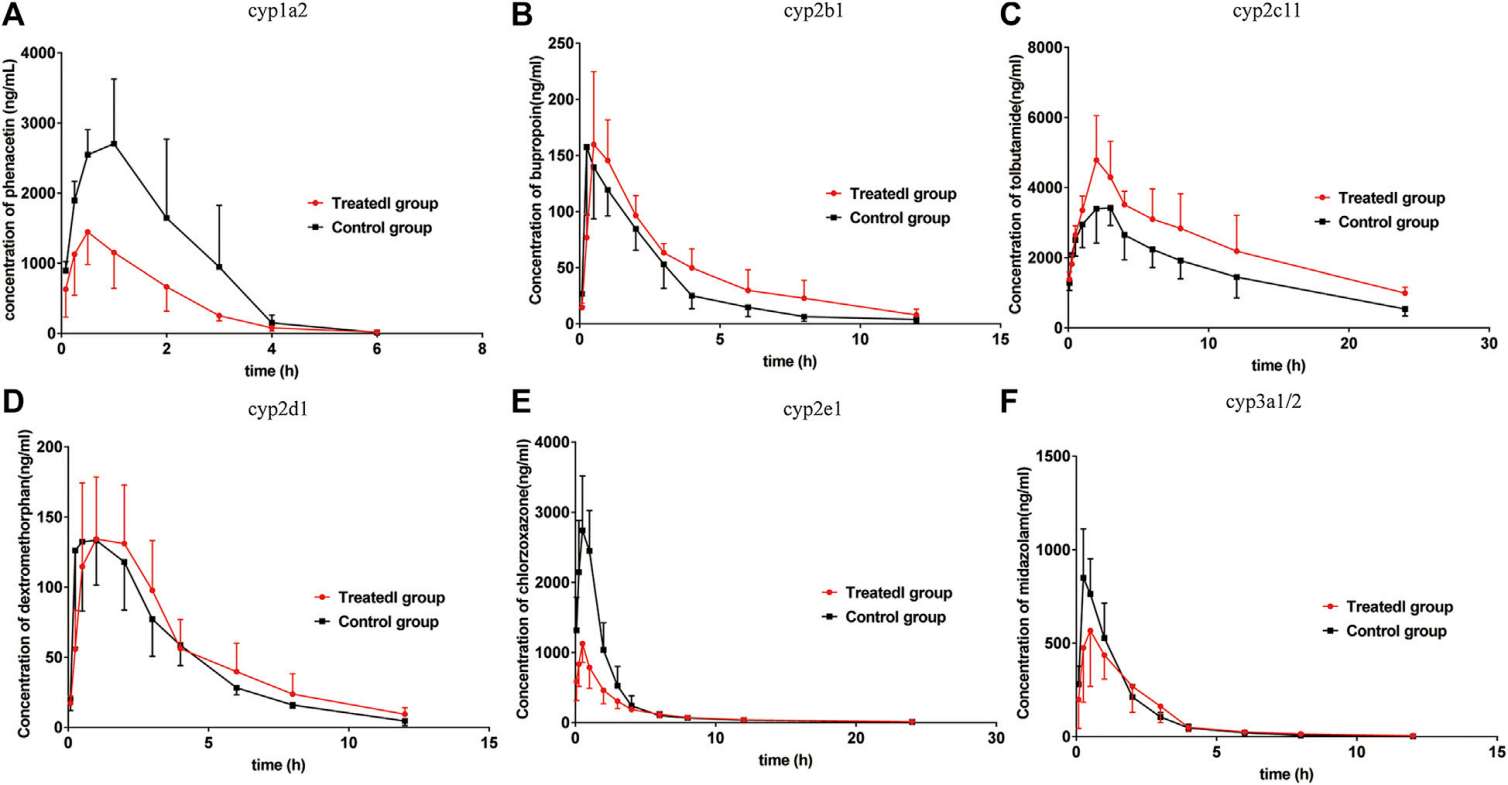
Mean plasma concentration-time curves of **(A)** phenacetin (CYP1A2), **(B)** bupropion (cyp2b1), **(C)** tolbutamide (cyp2c11), **(D)** dextromethorphan (cyp2d1), **(E)** chlorzoxazone (cyp2e1), and **(F)** midazolam (cyp3a1/2) in rats (mean ± SD, n = 6).

The pharmacokinetic parameters of phenacetin were used to describe the inhibitory effects of poziotinib on cyp1a2 activity. The area under the plasma concentration-time curve of phenacetin (Figure 5A) in the treated group was lower than that of the control group. The main pharmacokinetic parameters of phenacetin in the treated group were significantly different (*p* < 0.05) from those of the control group (Table 2). Compared with the control group, the AUC _(0–t)_, AUC _(0–∞)_, and C_max_ values of the treated group were significantly reduced by 56.08, 55.81, and 48.06%, respectively. In addition, t_1/2_, Vz/F, and CLz/F values were significantly increased by 45.45, 203.82, and 116.85%, respectively. The higher AUC and C_max_ values and lower T_max_ values of the control group indicated that poziotinib induces cyp1a2 activity, thus accelerating the metabolism of phenacetin and reducing its plasma drug concentration in rats.

**TABLE 2:** Primary pharmacokinetic parameters after oral administration of phenacetin and bupropion in rats.

Parameters	Phenacetin		Bupropion	
	Treated group	Control group	Treated group	Control group
AUC_(0–t)_ (µg/L∙h)	2,773.96 ± 1,019.57^a^	6,316.06 ± 2,956.76	564.82 ± 92.47^a^	404.774 ± 87.35
AUC_(0–∞)_ (µg/L∙h)	2,794.76 ± 1,016.11^a^	6,324.64 ± 2,953.20	613.41 ± 129.92^a^	421.07 ± 94.21
MRT_(0–t)_ (h)	1.41 ± 0.13	1.46 ± 0.22	3.33 ± 0.84^a^	2.37 ± 0.53
MRT_(0–∞)_ (h)	1.46 ± 0.15	1.46 ± 0.21	4.34 ± 1.58	2.87 ± 1.15
t_1/2_z (h)	0.80 ± 0.12^a^	0.55 ± 0.15	3.35 ± 1.21	2.51 ± 1.34
T_max_ (h)	0.54 ± 0.25	0.75 ± 0.27	0.75 ± 0.27	0.46 ± 0.29
Vz/F (L/kg)	4.77 ± 2.26^a^	1.57 ± 0.90	81.80 ± 38.30	85.39 ± 37.71
CLz/F (L/h∙kg)	3.99 ± 1.44^a^	1.84 ± 0.72	16.87 ± 3.27^a^	24.72 ± 5.29
C_max_ (µg/L)	1,491.60 ± 450.38^a^	2,869.22 ± 783.32	172.80 ± 60.83	170.12 ± 60.07

AUC, area under the plasma concentration–time curve; CL, plasma clearance, C_max_, maximum plasma concentration; MRT, mean residence time, t_1/2_, half-life; T_max_, time taken to reach maximum plasma level. n = 6 each group.

a Significantly different from control, *p* < 0.05.

### Effects of Poziotinib on the Activity of cyp2b1 in Rats

The mean plasma concentration-time curves of bupropion in the treated and control groups show that the AUC of the treated group was greater than that of the control group (Figure 5B). The AUC_(0–t)_, AUC_(0–∞),_ MRT_(0–t),_ and T_max_ values in the treated group were 1.39, 1.46, 1.41, and 1.63-fold higher than those of the control group, respectively, whereas the CLz/F and Vz/F values were 1.47 and 1.04-fold lower than those of the control group, respectively (Table 2). Thus, poziotinib reduced the metabolism of bupropion in rats, leading to its accumulation, which indicates that poziotinib had an inhibitory effect on cyp2b1 activity in rats.

### Effects of Poziotinib on the Activity of cyp2c11 in Rats

The AUC of tolbutamide in the treated group was higher than that of the control group (Figure 5C). AUC _(0–t)_ and AUC _(0–∞)_ values were significantly higher in the treated group than in the control group (*p* < 0.05), increasing by 45.87 and 51.60%, respectively, whereas CLz/F was significantly lower than control group by 50% (Table 3). Thus, we concluded that poziotinib had a significantly inhibitory effect on cyp2c11 activity in rats, leading to the accumulation of tolbutamide in rats.

**TABLE 3:** Primary pharmacokinetic parameters after oral administration of tolbutamide and dextromethorphan in rats.

Parameters	Tolbutamide		Dextromethorphan	
	Treated group	Control group	Treated group	Control group
AUC_(0–t)_ (µg/L∙h)	56,582.84 ± 14,772.74^a^	39,606.01 ± 9,276.12	640.28 ± 197.26	575.05 ± 77.66
AUC_(0–∞)_ (µg/L∙h)	75,199.99 ± 12,867.24^a^	49,603.73 ± 21,703.66	722.40 ± 231.03	586.36 ± 79.26
MRT_(0–t)_ (h)	8.88 ± 0.50	8.14 ± 0.69	3.54 ± 0.51	3.12 ± 0.37
MRT_(0–∞)_ (h)	17.91 ± 6.35	13.30 ± 5.86	5.19 ± 2.68	3.35 ± 0.47
t_1/2_z (h)	12.72 ± 4.84	9.10 ± 4.20	3.72 ± 2.09	1.98 ± 0.43
T_max_ (h)	2.33 ± 0.52	2.67 ± 0.52	1.58 ± 0.92	1.04 ± 0.56
Vz/F (L/kg)	0.25 ± 0.10	0.27 ± 0.04	75.51 ± 33.12	48.97 ± 10.71
CLz/F (L/h∙kg)	0.01 ± 0.00^a^	0.02 ± 0.01	15.01 ± 4.46	17.34 ± 2.54
C_max_ (µg/L)	4,922.08 ± 1,162.92	3,702.48 ± 870.99	152.96 ± 42.33	151.91 ± 57.66

AUC, area under the plasma concentration–time curve; CL, plasma clearance. C_max_, maximum plasma concentration; MRT, mean residence time; t_1/2_, half-life; T_max_, time taken to reach maximum plasma level. n = 6 each group.

a Significantly different from control, *p* < 0.05.

### Effects of Poziotinib on the Activity of cyp2d1 in Rats

There was no obvious difference in the average plasma concentration-time curves of dextromethorphan in the treated and control groups (Figure 5D). There were no significant differences in the main pharmacokinetic parameters of dextromethorphan such as AUC, C_max_, T_max_, t_1/2_, MRT, and CLz/F values between the two groups of rats (Table 3). In summary, poziotinib had no obvious inhibitory effect on cyp2d1 activity in rats.

### Effects of Poziotinib on the Activity of cyp2e1 in Rats

The AUC of chlorzoxazone in the treated group was lower than that of the control group (Figure 5E). The AUC_(0–t)_, AUC_(0–∞)_, and C_max_ values of the treated group were significantly reduced to 1.95, 1.94 and 2.51-fold that of the control group, respectively. The MRT _(0–t)_, MRT _(0–∞)_, Vz/F, and CLz/F of the treated group were 1.60, 1.56, 1.75, and 1.96-fold higher than those of the control group (Table 4). We concluded that poziotinib induced cyp2e1 activity and accelerated the metabolism of chlorzoxazone in rats.

**TABLE 4:** Primary pharmacokinetic parameters after oral administration of chlorzoxazone and midazolam in rats.

Parameters	Chlorzoxazone		Midazolam	
	Treated group	Control group	Treated group	Control group
AUC_(0–t)_ (µg/L∙h)	3,150.75 ± 812.16^a^	6,137.83 ± 1,445.32	1,277.30 ± 500.56	1,351.70 ± 298.80
AUC_(0–∞)_ (µg/L∙h)	3,200.40 ± 846.30^a^	6,205.45 ± 1,492.39	1,308.05 ± 494.28	1,356.60 ± 300.09
MRT_(0–t)_ (h)	3.87 ± 0.55^a^	2.42 ± 0.28	2.01 ± 0.43	1.58 ± 0.23
MRT_(0–∞)_ (h)	4.24 ± 0.74^a^	2.72 ± 0.46	2.44 ± 1.12	1.62 ± 0.24
t_1/2_z (h)	4.36 ± 1.17	5.00 ± 2.33	2.67 ± 1.39	1.70 ± 0.55
T_max_ (h)	0.50 ± 0.00	0.67 ± 0.26	0.58 ± 0.34	0.33 ± 0.13
Vz/F (L/kg)	19.76 ± 2.25^a^	11.32 ± 3.64	32.83 ± 20.76	18.62 ± 5.95
CLz/F (L/h∙kg)	3.36 ± 1.06^a^	1.71 ± 0.53	8.34 ± 2.28	7.67 ± 1.64
C_max_ (µg/L)	1,127.01 ± 267.71^a^	2,833.44 ± 777.71	581.77 ± 296.06	868.27 ± 250.7

AUC, area under the plasma concentration–time curve; CL, plasma clearance; C_max_, maximum plasma concentration; MRT, mean residence time; t_1/2_, half-life; T_max_, time taken to reach maximum plasma level. n = 6 each group.

a Significantly different from control, *p* < 0.05.

### Effects of Poziotinib on the Activity of cyp3a1/2 in Rats

The AUC of midazolam was lower in the treated group than in the control group (Figure 5F). The AUC and C_max_ values of the control group were higher than those of the treated group, whereas the CLz/F value of the treated group was higher than that of the control group (Table 4), but these differences were not significant. Therefore, we concluded that poziotinib had no significant inhibitory effect on cyp3a1/2 activity in rats.

## Discussion

This study is the first to examine the induction/inhibition effects of poziotinib on CYP enzyme activity in RLMs by using six substrate probes and investigate the interactions of poziotinib with the probe substrates by using the cocktail method with RLMs as well as in rats.

The results of *in vitro* incubation with RLMs showed that IC_50_ (bupropion) > IC_50_ (tolbutamide) > IC_50_ (dextromethorphan) > IC_50_ (phenacetin) > IC_50_ (chlorzoxazone) > IC_50_ (midazolam). According to literature, IC_50_ < 1 μM is considered to be strong inhibition, 1 μM < IC_50_ < 10 μM moderate inhibition, and IC_50_ > 10 μM weak inhibition (Jin et al., 2015). Our results show that poziotinib has moderate and weak inhibitory effects on CYP2B1 (IC_50_ = 8.79 μM) and CYP2C11 (IC_50_ = 20.17 μM), respectively, whereas its inhibitory effect on the other four CYPs was not obvious (IC_50_ of cyp1a2, cyp2d1, cyp2e1 and cyp3a1/2 were 128.2, 61.96, 156.3, and 302.7 μM, respectively). A more detailed investigation of the inhibitory effect of poziotinib on cyp2b1 and 2c11 revealed that the inhibition of both cyp2b1 and 2c11 by poziotinib was competitive, with Ki values of 16.18 and 17.66 μM, respectively.

As the approved EGFR inhibitor, poziotinib is a quinazoline derivative, same as afatinib, erlotinib, gefitinib, dacomitinib. The above quinazoline derivatives are all metabolized by CYP 2D6 and CYP 3A4, however, they have different impacts on CYPs activity. Afatinib has no inhibition or induction on cytochrome P450 (CYP) enzymes (Dungo et al., 2013). Erlotinib may inhibit the activity of cyp2b1 and cyp3a1/2 in rats (Ma et al., 2015), and inhibit warfarin metabolism, a CYP2C9 substrate, in human (Hiraide et al., 2019). Gefitinib not only inhibit CYP2C9, but also CYP2D6 (Prommer, 2004; Hiraide et al., 2019). A similar, dacomitinib is a potent inhibitor of CY2D6 (Shirley, 2018). In our study, poziotinib inhibits cyp2b1 and cyp2c11 in RLM and in rats with competitive model, and induces cyp1a2 and cyp2e1 activities in rats.

This competitive inhibition indicates that the inhibitor, poziotinib, and the enzyme substrate compete at the binding site, thereby interfering with the binding of the substrate to the enzyme and reducing enzymatic activity. In addition, we did not observe any time- and concentration-dependent inhibitory effects of poziotinib on cyp2b1 and 2c11 in RLMs, which implies that the activity of these enzymes did not change over time during the incubation period. These results indicate that cyp2b1 and cyp2c11 were reversibly inhibited by poziotinib. Therefore, attention should be paid to the combined use of poziotinib and drugs to look for possible interactions of poziotinib with drugs that are metabolized by cyp2b1 or cyp2c11 to prevent the occurrence of serious adverse reactions.

We compared pharmacokinetic parameters of the six CYP probe substrates as a result of their interaction with poziotinib in rats. For cyp2b1 and bupropion, the AUC_(0–t)_ and CLz/F values of the treated group were 1.40 and 0.68-fold that of the control group values, respectively. Similarly, for cyp2c11 and tolbutamide, the AUC_(0–t)_ and CLz/F values of the treated group were 1.43 and 0.5-fold that of the control group values, respectively. These results indicate that the activity of cyp2b1 and 2c11 was inhibited by poziotinib, which resulted in increased systemic exposure of bupropion and tolbutamide in rats and slower clearance rates. This was consistent with the results of the *in vitro* incubation with RLMs, confirming the inhibitory effect of poziotinib on the activity of cyp2b1 and 2c11.

This inhibition could be attributed to the direct binding of poziotinib to the substrate binding site in the enzymes, leading to a decrease in the metabolic activity of these two enzymes. Another study had postulated that long-term use of poziotinib may affect the expression of metabolic enzymes (Wang et al., 2018). In addition, we found that poziotinib induced cyp1a2 and cyp2e1 activity with the two probe substrates, phenacetin and chlorzoxazone, respectively, because the AUC and C_max_ values in the treated groups were significantly lower than those in the respective control groups, whereas CLz/F values were much higher than those in the control groups. Thus, poziotinib accelerated the elimination rates and reduced the plasma concentrations of phenacetin and chlorzoxazone in rats. However, our *in vitro* results did not reveal any inhibitory effect of poziotinib on cyp1a2 and 2e1. This could imply that the observed induction in rats may be caused by gene regulation, which increased protein synthesis and accelerated the metabolism of the probe substrates; however, further research is needed to confirm this hypothesis (Yan, 2012). Poziotinib had no inhibitory effect on the enzymatic activity of cyp2d1 and cyp3a1/2 *in vivo* or *in vitro*.

## Conclusion

In conclusion, this study shows that poziotinib competitively and reversibly inhibited the metabolism of cyp2b1 and cyp2c11 substrates to varying degrees without any time- or concentration dependence. Poziotinib had no obvious inhibitory effect on the other four CYPs. The results of this study will help in further study of poziotinib in humans and draw attention to its potential DDIs when combined with other drugs metabolized by cyp2b1 and cyp2c11 to enable clinically effective and safe use of poziotinib.

## Data Availability

The original contributions presented in the study are included in the article/Supplementary Material, further inquiries can be directed to the corresponding authors.
