# Supplementary material for: Inhibition and Induction by Poziotinib of Different Rat Cytochrome P450 Enzymes In Vivo and in an In Vitro Cocktail Method
Source: Front Pharmacol. 2021 Jan 5;11:593518. doi: 10.3389/fphar.2020.593518 (PMC7970697; doi:10.3389/fphar.2020.593518)
Supplement: Supplementary file 1 [file datasheet1.pdf]

Inhibition and Induction by Poziotinib of Different Rat Cytochrome P450 Enzymes *In Vivo* and in an *In Vitro* Cocktail Method

Supplementary Materials

Figure S1. Chemical structure of (**A**) phenacetin, (**B**) bupropion, (**C**) tolbutamide, (**D**) dextromethorphan, (**E**) chlorzoxazone, (**F**) midazolam, and (**G**) diazepam (internal standard, IS).

Figure S2. Typical multiple reaction monitoring (MRM) chromatograms of (A) chlorzoxazone, (B) midazolam, (C) diazepam , (D) dextromethorphan, (E) tolbutamide, (F) bupropion, (G) phenacetin and (H) TIC in plasma samples.

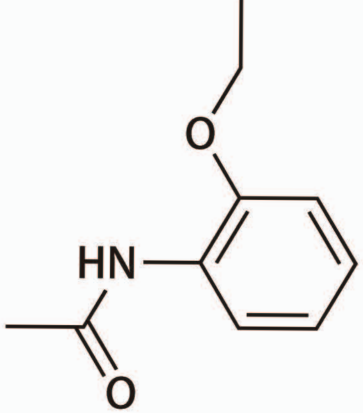

(A) Phenacetin

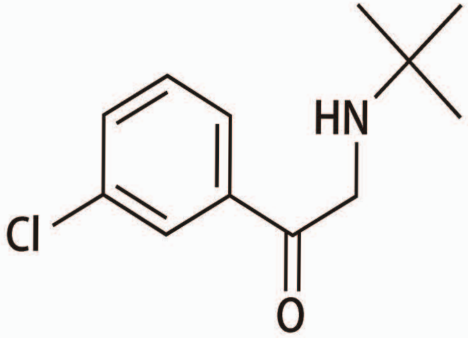

(B) Bupropion

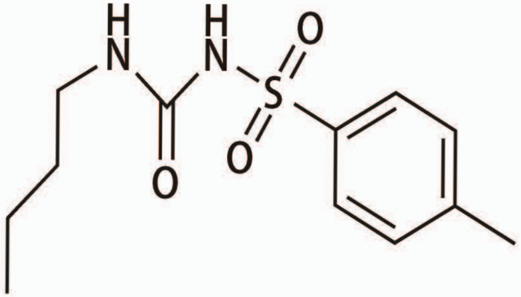

(C) Tolbutamide

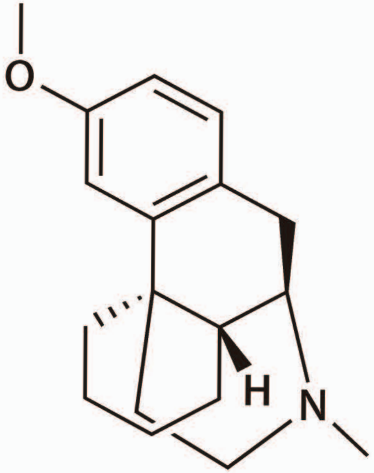

(D) Dextromethophan

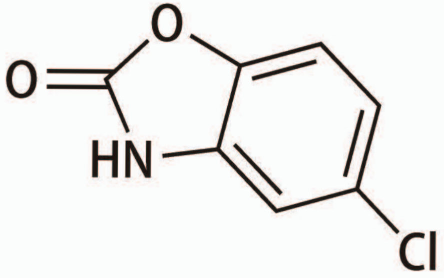

(E) Chlorzoxazone

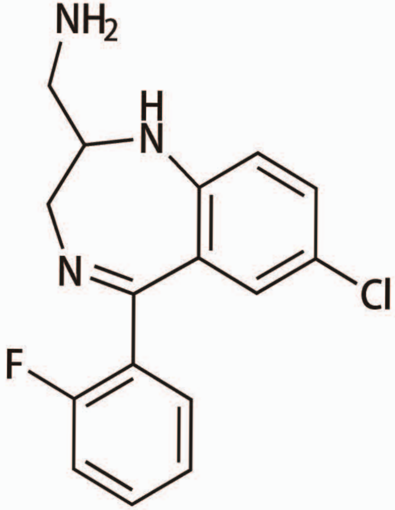

(F) Midazolam

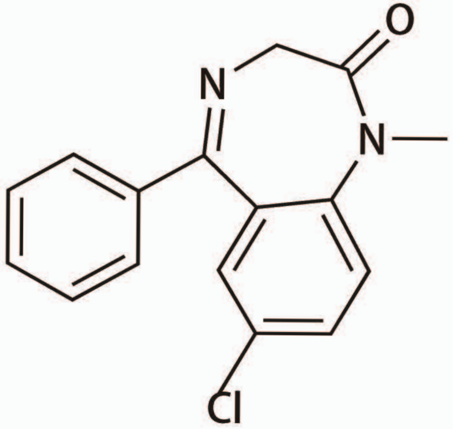

(G) Diazepam (IS)

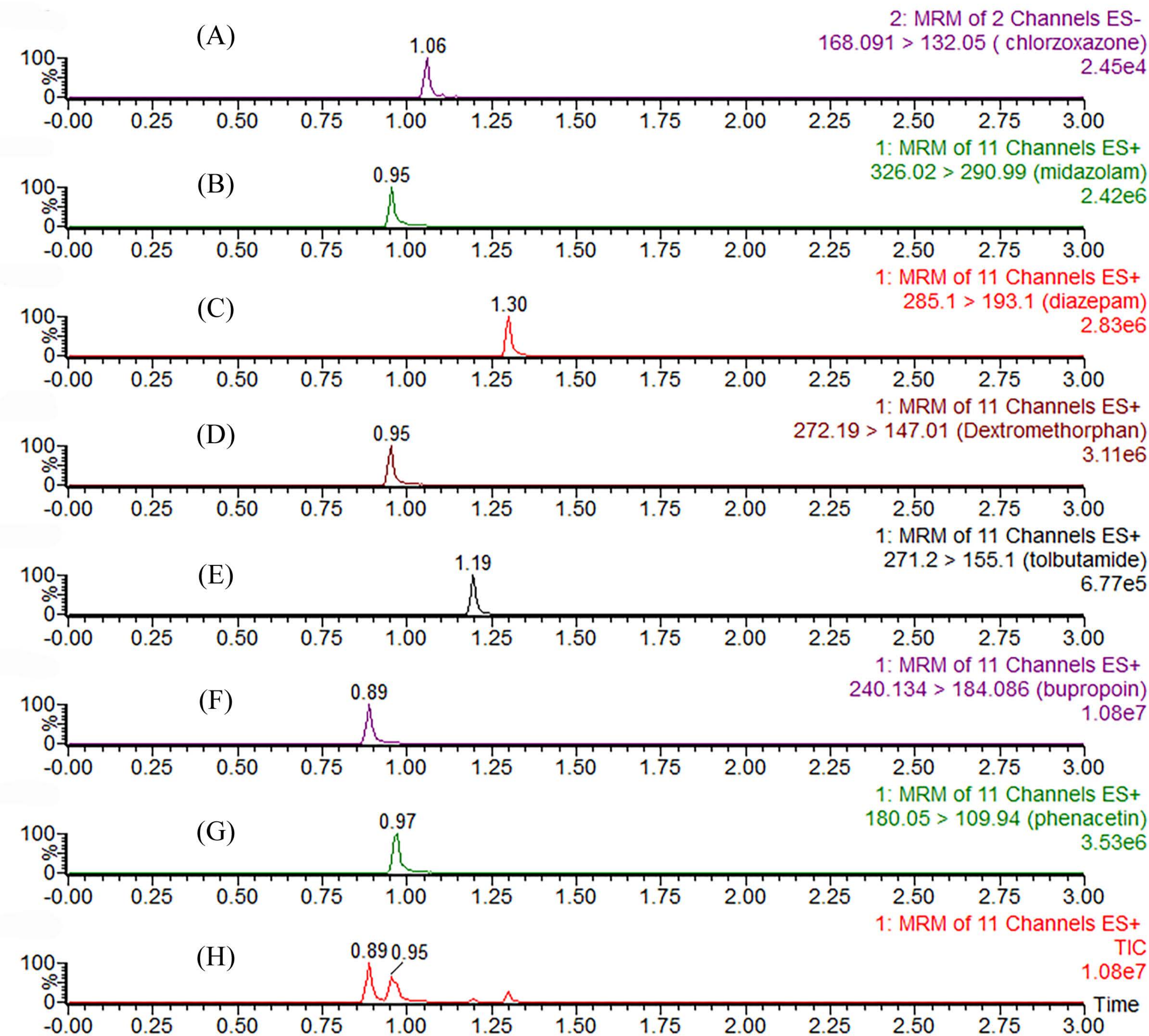
(A)
2: MRM of 2 Channels ES-
168.091 > 132.05 (chlorzoxazone)
2.45e4
1.06
(B)
1: MRM of 11 Channels ES+
326.02 > 290.99 (midazolam)
2.42e6
0.95
(C)
1: MRM of 11 Channels ES+
285.1 > 193.1 (diazepam)
2.83e6
1.30
(D)
1: MRM of 11 Channels ES+
272.19 > 147.01 (Dextromethorphan)
3.11e6
0.95
(E)
1: MRM of 11 Channels ES+
271.2 > 155.1 (tolbutamide)
6.77e5
1.19
(F)
1: MRM of 11 Channels ES+
240.134 > 184.086 (bupropoin)
1.08e7
0.89
(G)
1: MRM of 11 Channels ES+
180.05 > 109.94 (phenacetin)
3.53e6
0.97
(H)
1: MRM of 11 Channels ES+
TIC
1.08e7
0.89
0.95
Time
100
%
0
-0.00
0.25
0.50
0.75
1.00
1.25
1.50
1.75
2.00
2.25
2.50
2.75
3.00
